# Esophago-bronchial fistula treated by the Over-The-Scope-Clipping (OTSC) system with argon beam electrocoagulation: A case report

**DOI:** 10.1097/MD.0000000000024494

**Published:** 2021-01-29

**Authors:** Jun Sonomura, Tetsunosuke Shimizu, Kohei Taniguchi, Sang-Woong Lee, Ryo Tanaka, Yoshiro Imai, Kotaro Honda, Masaru Kawai, Keitaro Tashiro, Kazuhisa Uchiyama

**Affiliations:** a Department of General and Gastroenterological Surgery; b Translational Research Program, Osaka Medical College, 2-7, Daigaku-machi, Takatsuki city, Osaka, Japan.

**Keywords:** argon beam electrocoagulation, esophageal bronchial fistula, esophageal cancer, Over-The-Scope-Clipping

## Abstract

**Rationale:**

An esophago-bronchial fistula is one of the rare postoperative complications of esophageal cancer. There are various medical treatments, including suturing, endoscopic clip, and fibrin glue. However, these treatments often lead to unsatisfactory results, causing physicians to opt for surgical alternatives. The Over-The-Scope-Clipping (OTSC) system offers an alternative method for fistula closure. It can capture a large amount of tissue and is able to compress the lesion until it has fully healed. However, data indicating the efficacy of OTSC for esophago-bronchial fistula are limited.

**Patient concerns:**

A 64-year-old man presented with an esophago-bronchial fistula after surgery for esophageal cancer. We chose to use a stent as the first line of treatment, but the fistula did not close.

**Diagnoses:**

Intractable esophago-bronchial fistula associated with esophageal surgery.

**Interventions and Outcomes:**

On the 94th postoperative day, fistula closure with OTSC was performed, and no leakage of the contrast agent was observed during fluoroscopy. We also attempted to close the fistula by combining OTSC and argon plasma coagulation (APC) to burn off the scar tissue from around the fistula. The fistula gradually shrank after a total of 4 rounds of OTSC, and closure of the fistula was achieved on the 185th postoperative day. There were no adverse events during the treatment of this case.

**Lessons:**

We demonstrate that OTSC is useful in the management of esophago-bronchial fistulas, and may become a standard procedure for the endoscopic treatment of esophago-bronchial fistulas, replacing the use of stents, clips, or glue.

## 1 Introduction

Postoperative complications of esophageal cancer are often life-threatening. An esophageal bronchial fistula is one of the rare postoperative complications of esophageal cancer, affecting approximately 1% of these patients.^[1]^ Early diagnosis can help in avoiding severe pulmonary complications, and early intervention can improve the chances of cure. Non-surgical treatment options, such as suturing,^[2]^ endoscopic clips,^[3]^ and fibrin glue,^[4]^ are contraindicated for esophageal bronchial fistulas due to unsatisfactory results. As such, surgical treatment is frequently selected because of insufficiencies associated with non-surgical treatments. The Over-The-Scope-Clipping (OTSC; Ovesco, Tuebingen, Germany) system is a clipping system used in endoscopy. The system is indicated for gastrointestinal bleeding, gastrointestinal perforations and fistulas, and complications of endoscopic and surgical procedures.^[5–7]^ The clip is placed on the tissue in the cap, and fistula closure is completed by using endoscopic suction to pull the tissue around the fistula into the cap. There are limited data on the use of the OTSC system for closing esophageal bronchial fistulas. We report a case in which a fistula was successfully closed using the OTSC system.

## 2 Case presentation

A 64-year-old man was diagnosed with advanced esophageal cancer (Mt, T2, N1, M0, Stage II) by upper endoscopy after the chief complaint of pharyngeal discomfort. There were no remarkable blood analysis findings. Tumor markers such as carcinoembryonic antigen, carbohydrate antigen 19–9, and squamous cell carcinoma antigen were within the normal range, and the cytokeratin fragment 21–1 level was elevated, at 4.9 ng/mL. After neoadjuvant chemotherapy (5-fluorouracil and cisplatin), the patient underwent thoracoscopic subtotal esophagectomy, laparoscopic gastric tube reconstruction, and cervical thoraco-abdominal three-field lymph node dissection. An anastomosis of the esophagus and gastric tube was performed using the triangular anastomotic method, through the posterior mediastinal route, via a 45-mm diameter automatic anastomosis instrument. The total operative time was 9 hours, and the total blood loss was 30 mL. The histopathological findings revealed esophageal cancer (Mt, after chemotherapy, CT-ypT0 [T1b], ypN0, CRT-Grade 3, Stage 0) (Esophageal Cancer Treatment Agreement, 11th edition).

**Figure 1:**
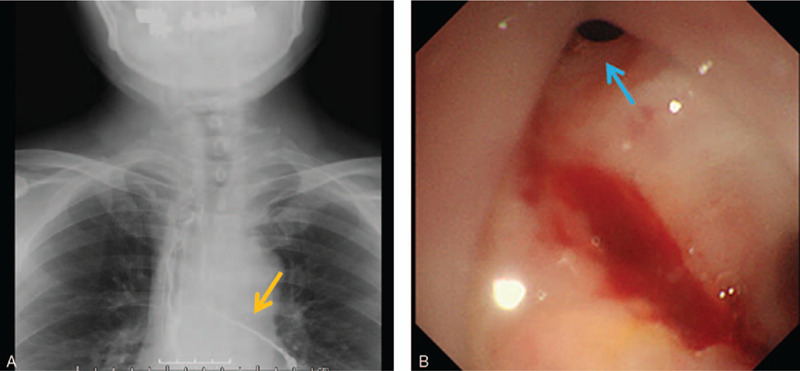
The representative findings of esophago-bronchial fistula are shown. (A) Gastrografin contrast study shows leakage into the left bronchus (orange arrow). (B) A fistula is detected in the anterior wall of the esophagogastric anastomotic area by endoscopy (blue arrow).

The patient did not resume oral food intake until the 6th postoperative day (POD), leading to a delay in the recognition of an apparent anastomotic leak. He developed a fever after starting meal ingestion, and we subsequently diagnosed the patient with anastomotic leakage by enhanced computed tomography examination. The patient was returned to jejunostomy-tube feeding for 3 weeks, causing the inflammation to subside gradually. Since a slight fever and cough persisted, fluoroscopy and upper endoscopy were performed on the 37th POD (Fig. 1A and B), and we diagnosed an esophago-bronchial fistula. A stent was inserted on the 42nd POD. The stent position was appropriately adjusted and left in situ for approximately 3 weeks, but the fistula did not close. After that, despite concomitant use of an endoscopic clip and a stent for about a month (25 days), the fistula did not close. We concluded that fistula closure with a stent was not viable (total stenting duration was 50 days), and decided to use the OTSC system (Ovesco Endoscopy AG, Germany).

**Figure 2:**
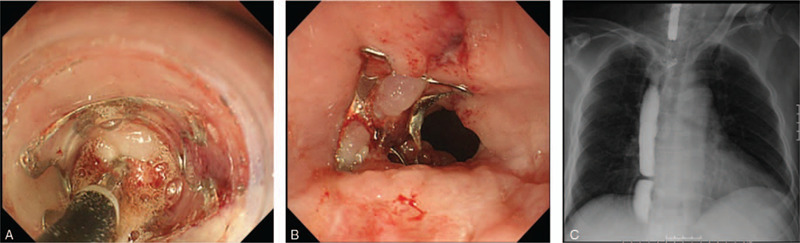
Representative images of Over-The-Scope-Clipping (OTSC) procedures are shown. (A) Grasping of the fistula by a gripping tool. (B) The first round of fistula closure by OTSC. (C) Gastrografin contrast study shows no contrast material into the bronchus. Fistula closure by OTSC (third round) is shown here.

**Figure 3:**
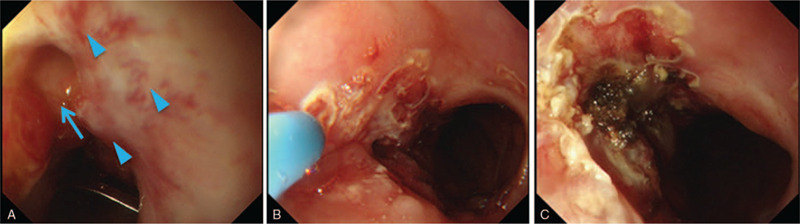
Representative images of the scar around the fistula before completion of Over-The-Scope-Clipping (OTSC) procedures. (A) A scar is found around the fistula, resulting in difficulty in clipping (blue arrowheads). Blue arrow indicates the fistula. (B): Removal of the scar by argon plasma coagulation. (C): OTSC procedures were performed after removal of the scar.

**Figure 4:**
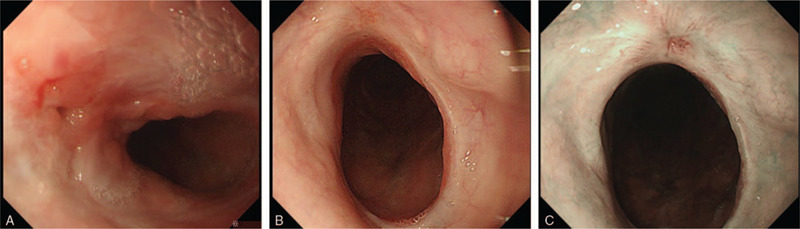
Representative images of the fistula and scar after Over-The-Scope-Clipping (OTSC) with argon plasma coagulation (APC). (A) The size of the fistula is decreased. (B) The fistula has healed, with a clear scar. (C) Narrow-band imaging of (B).

**Figure 5:**
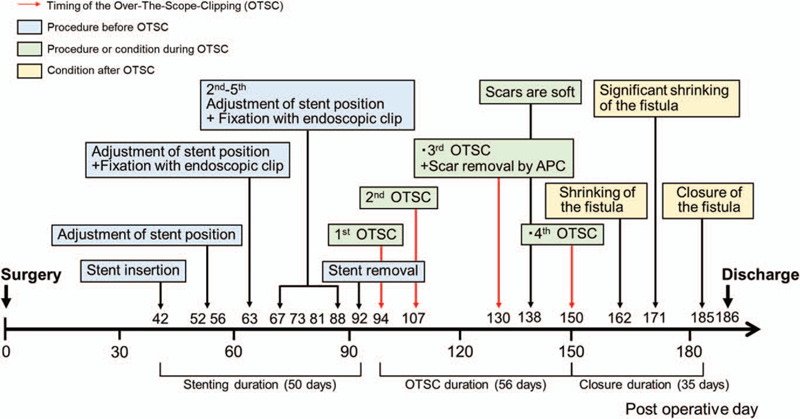
Summary of the procedures and postoperative course. The summary of the patient's course focuses on the endoscopic findings and procedures. The red arrows indicate the timing of OTSC rounds. Blue boxes indicate the timings of stent-related procedures completed before OTSC. Green boxes indicate the OTSC round number and condition. Yellow boxes indicate the findings of the fistula after completion of the OTSC procedures. APC = argon plasma coagulation, OTSC = Over-The-Scope-Clipping, POD = postoperative day.

The t type (traumatic) clip of the OTSC system (Ovesco Endoscopy AG) was selected. Initially, the clip width was 10 mm. On the 94th POD, fistula (3–4 mm) closure with the OTSC system was performed after removal of the stent (Fig. 2A and B), and no leakage of the contrast agent was observed during fluoroscopy (Fig. 2C). Five weeks after the first OTSC procedure (during the third OTSC procedure), due to insufficient results, we attempted to close the fistula by combining the OTSC system (Fig. 3A–C) and argon plasma coagulation (APC). APC was performed as scarring around the fistula was hindering the clipping. At this point, a fistula of 1 mm in size was observed. APC was therefore used to burn off the scar tissue from around the fistula before the OTSC procedure (VIO300D/APC2, Erbe Elektromedizin GmbH, Germany, Mode; forced, Output; 30–35 W, Argon flow 1.3 L/min). The fistula gradually shrank after OTSC was performed for a total of 4 times (56 d/8 wks) (Fig. 4A). During the first and the second OTSC procedure, a 10 mm clip was used. For the third and fourth OTSC procedures, the clip was down-sized to 9 mm. The fistula's closure was confirmed by contrast-enhanced computerized tomography (CT) on the 183rd POD and upper endoscopy on the 185th POD (35 d/5 wks after the last OTSC) (Fig. 4B and C). The postoperative course is summarized in Fig. 5. The patient was discharged the following day, and follow up examinations included contrast-enhanced CT every 6 months and upper endoscopy which were performed annually. No recurrence or complications have been reported to date (6 years have passed in 2020). We plan to perform follow up evaluations with similar tests annually in the future.

## 3 Discussion

In the present report, we described a case in which an esophago-bronchial fistula was successfully closed using the OTSC system. In general, the first choice for the treatment of esophageal fistula is to use a retrievable, self-expanding, plastic stent.^[8]^ Indeed, we initially used a HANAROSTENT Esophagus stent (Olympus, Tokyo, Japan) that has specialized features including dumbbell-shaped heads on both ends to prevent stent migration by esophageal peristalsis, kink resistance, a backflow prevention valve, and lassos on both ends for easy adjustment of stent positioning. The success rate of using a plastic stent to treat an esophageal fistula after esophagectomy ranges from 67% to 100%.^[8–13]^ However, the following disadvantages of using a plastic stent for treating esophageal fistulas must be considered: migration of the stent; long-term detention; stenosis of the esophagus; tissue necrosis due to stent pressure; and obstruction due to ulceration and granulation.

OTSC was developed in 2007 for endoscopic closure during natural orifice transluminal endoscopic surgery.^[14]^ With recent advances in technological developments, the efficacy of OTSC as a therapeutic option has mainly been reported for the management of gastrointestinal perforation.^[5,15,16]^ This technique has been found to achieve high rates of adequate closure and low rates of postoperative leakage, and allows for simple and fast operations, as described in the multicenter CLIPPER study.^[6]^ According to this report, adequate closure was achieved in 89% of cases within about 5 minutes, and only one patient underwent surgical intervention. Other reports have also described high clinical efficacy rates and adequate closure rates (64%–100%).^[5–7,16]^ In terms of complications associated with the use of OTSC, stenosis of the esophagus, perforation by the claw of the clip, aggravation of the primary lesion, and migration of the clip have been reported. However, the serious complication rate is very low.^[5–7,16–20]^ Comprehensively, OTSC may be recognized as a device with a high level of safety and permissible complication rates.

Numerous reports exist on therapeutic interventions for various fistulas, including rectovaginal fistula,^[21]^ recto-acetabular fistula,^[22]^ and fistula associated with esophagus and bronchial tubes.^[23]^ However, there is limited information available regarding the use of OTSC for closure of esophago-bronchial fistula after esophagectomy for patients with esophageal cancer. In our case, we decided to use OTSC because closure of the fistula by stent had failed. The success rate for fistula closure using OTSC has been reported to be low, at about 50% (33%–77%).^[5,7,18–20]^ Furthermore, it has been reported that the clinical success rate of OTSC for closure of acute fistula (persisting for <30 days) is 86%, while for chronic fistula (persisting for >30 days), the success rate was just 33%.^[7]^ Moreover, the overall long-term clinical success was lowest for fistulae (42.9%), followed by leakages (73.3%), and then perforations (90%).^[19]^ Further, 46% of patients who underwent OTSC developed fistula recurrence at a median of 39 days.^[19]^

The reason behind the lower success rate of OTSC for fistula compared with that for perforation or bleeding may be that the formation of scar tissue around the fistula during the healing process hinders the closure of the fistula. The key process in the use of OTSC is the use of a vacuum to pull the whole fistula into the cap attached to the tip of the fiberscope. The weight of this tissue will inevitably place more pressure on the clip. On the other hand, the success rate of OTSC for bleeding and perforation is likely to be higher because the tissue around the lesion is not fibrotic, and is, therefore, softer and easier to clip. Although our experience comprises a single case, we propose that the application of OTSC, as a primary treatment, may be a very effective option for esophago-bronchial fistula, but as a secondary treatment, the success rate of OTSC, with or without APC,^[24]^ is low.

In conclusion, we encountered a case in which OTSC was useful in the management of an esophago-bronchial fistula. OTSC was performed easily and safely, without adverse events. OTSC may become a standard procedure for endoscopic treatment of esophageal tracheal fistulas, replacing the use of stents, clips, or glue.

## Acknowledgments

The authors thank all staff of Osaka Medical College for their aid in the management of the patient. They also appreciate the work performed by Mr. Shinya Abe (Endoscopy Center, Osaka Medical College Hospital). And would also like to thank Editage (www.editage.com) for English language editing.

## Author contributions

**Conceptualization:** Jun Sonomura, Kohei Taniguchi, Tetsunosuke Shimizu.

**Data curation:** Kohei Taniguchi, Sang-Woong Lee.

**Investigation:** Ryo Tanaka, Yoshiro Imai, Kotaro Honda, Masaru Kawai, Keitaro Tashiro, Kazuhisa Uchiyama.

**Resources:** Sang-Woong Lee, Masaru Kawai, Keitaro Tashiro.

**Supervision:** Kazuhisa Uchiyama.

**Visualization:** Jun Sonomura, Kohei Taniguchi, Tetsunosuke Shimizu.

**Writing – original draft:** Jun Sonomura.

**Writing – review & editing:** Kohei Taniguchi, Tetsunosuke Shimizu, Kazuhisa Uchiyama.

## Abbreviations

Abbreviations: APC = argon plasma coagulation, OTSC = Over-The-Scope-Clipping, POD = postoperative day.

## References

[1] Lambertz R Hölscher AH Bludau M. Management of tracheo- or bronchoesophageal fistula after Ivor-Lewis esophagectomy. World J Surg 2016;40:1680–7. 26913731 10.1007/s00268-016-3470-9

[2] Bonin EA Wong Kee Song LM Gostout ZS. Closure of a persistent esophagopleural fistula assisted by a novel endoscopic suturing system. Endoscopy 2012;44:E8–9. 22396292 10.1055/s-0031-1291494

[3] Raymer GS Sadana A Campbell DB. Endoscopic clip application as an adjunct to closure of mature esophageal perforation with fistulae. Clin Gastroenterol Hepatol 2003;1:44–50. 15017516 10.1053/jcgh.2003.50007

[4] Rábago LR Ventosa N Castro JL. Endoscopic treatment of postoperative fistulas resistant to conservative management using biological fibrin glue. Endoscopy 2002;34:632–8. 12173084 10.1055/s-2002-33237

[5] Kirschniak A Subotova N Zieker D. The Over-The-Scope Clip (OTSC) for the treatment of gastrointestinal bleeding, perforations, and fistulas. Surg Endosc 2011;25:2901–5. 21424197 10.1007/s00464-011-1640-2

[6] Voermans RP Le Moine O von Renteln D. Efficacy of endoscopic closure of acute perforations of the gastrointestinal tract. Clin Gastroenterol Hepatol 2012;10:603–8. 22361277 10.1016/j.cgh.2012.02.005

[7] Haito-Chavez Y Law JK Kratt T. International multicenter experience with an over-the-scope clipping device for endoscopic management of GI defects (with video). Gastrointest Endosc 2014;80:610–22. 24908191 10.1016/j.gie.2014.03.049

[8] Hünerbein M Stroszczynski C Moesta KT. Treatment of thoracic anastomotic leaks after esophagectomy with self-expanding plastic stents. Ann Surg 2004;240:801–7. 15492561 10.1097/01.sla.0000143122.76666.ae PMC1356485

[9] Schubert D Scheidbach H Kuhn R. Endoscopic treatment of thoracic esophageal anastomotic leaks by using silicone-covered, self-expanding polyester stents. Gastrointest Endosc 2005;61:891–6. 15933696 10.1016/s0016-5107(05)00325-1

[10] Langer FB Wenzl E Prager G. Management of postoperative esophageal leaks with the Polyflex self-expanding covered plastic stent. Ann Thorac Surg 2005;79:398–403. discussion 404. 15680802 10.1016/j.athoracsur.2004.07.006

[11] Gelbmann CM Ratiu NL Rath HC. Use of self-expandable plastic stents for the treatment of esophageal perforations and symptomatic anastomotic leaks. Endoscopy 2004;36:695–9. 15280974 10.1055/s-2004-825656

[12] Doniec JM Schniewind B Kahlke V. Therapy of anastomotic leaks by means of covered self-expanding metallic stents after esophagogastrectomy. Endoscopy 2003;35:652–8. 12929059 10.1055/s-2003-41509

[13] Roy-Choudhury SH Nicholson AA Wedgwood KR. Symptomatic malignant gastroesophageal anastomotic leak: management with covered metallic esophageal stents. Am J Roentgenol 2001;176:161–5. 11133560 10.2214/ajr.176.1.1760161

[14] Kirschniak A Kratt T Stüker D. A new endoscopic over-the-scope clip system for treatment of lesions and bleeding in the GI tract: first clinical experiences. Gastrointest Endosc 2007;66:162–7. 17591492 10.1016/j.gie.2007.01.034

[15] Gubler C Bauerfeind P. Endoscopic closure of iatrogenic gastrointestinal tract perforations with the over-the-scope clip. Digestion 2012;85:302–7. 22614286 10.1159/000336509

[16] Hagel AF Naegel A Lindner AS. Over-the-Scope Clip application yields a high rate of closure in gastrointestinal perforations and may reduce emergency surgery. J Gastrointest Surg 2012;16:2132–8. 22903364 10.1007/s11605-012-1983-6

[17] Baron TH Song LM Ross A. Use of an over-the-scope clipping device: multicenter retrospective results of the first U.S. experience (with videos). Gastrointest Endosc 2012;76:202–8. 22726484 10.1016/j.gie.2012.03.250

[18] Surace M Mercky P Demarquay J-F. Endoscopic management of GI fistulae with the over-the-scope clip system (with video). Gastrointest Endosc 2011;74:1416–9. 22136786 10.1016/j.gie.2011.08.011

[19] Law R Wong Kee Song LM Irani S. Immediate technical and delayed clinical outcome of fistula closure using an over-the-scope clip device. Surg Endosc 2015;29:1781–6. 25277480 10.1007/s00464-014-3860-8

[20] Sulz MC Bertolini R Frei R. Multipurpose use of the over-the-scope-clip system (“Bear claw”) in the gastrointestinal tract: Swiss experience in a tertiary center. World J Gastroenterol 2014;20:16287–92. 25473185 10.3748/wjg.v20.i43.16287 PMC4239519

[21] Tong Y Trilling B Sage PY. Short-term outcomes of the over-the-scope clip proctology system for rectovaginal fistula repair: a prospective study. Tech Coloproctol 2019;23:245–9. 30937645 10.1007/s10151-019-01948-5

[22] Schiergens TS Becker CC Weber P. Over-the-scope clip (OTSC®) closure of a recto-acetabular fistula. J Surg Case Rep 2018;4:1–3. 10.1093/jscr/rjy074 PMC5915952 29713447

[23] Balakrishnan A Tapias L Wright CD. Surgical management of post-esophagectomy tracheo-bronchial-esophageal fistula. Ann Thorac Surg 2018;106:1640–6. 30171850 10.1016/j.athoracsur.2018.06.076

[24] van Bodegraven AA Kuipers EJ Bonenkamp HJ. Esophagopleural fistula treated endoscopically with argon beam electrocoagulation and clips. Gastrointest Endosc 1999;50:407–9. 10462666 10.1053/ge.1999.v50.97234

